# Knowledge, Attitudes, Practices, and Predictors of Anaemia Among Pregnant Women Attending Antenatal Care at a District Hospital in Ghana: A Cross-Sectional Study

**DOI:** 10.1155/anem/7333987

**Published:** 2025-06-05

**Authors:** Richard Vikpebah Duneeh, Hansen Benjamin Tetteh, Mercy Adzo Klugah, Memunatu Jibrim, Richard Otu Agblom, Precious Kwablah Kwadzokpui, Kenneth Ablordey

**Affiliations:** ^1^Department of Medical Laboratory Sciences, School of Allied Health Sciences, University of Health and Allied Sciences, Ho, Ghana; ^2^Department of Medical Diagnostics, College of Health Sciences, Kwame Nkrumah University of Science and Technology, Kumasi, Ghana; ^3^Department of Medical Laboratory, Catholic Hospital, Battor, Ghana; ^4^Department of General and Liberal Studies, School of Basic and Biomedical Sciences, University of Health and Allied Sciences, Ho, Ghana; ^5^Department of Epidemiology and Disease Control, School of Public Health, University of Ghana, Accra, Ghana

**Keywords:** anaemia, haemoglobin, pregnancy, trimester

## Abstract

**Background:** Anaemia during pregnancy is a major public health concern in both advanced and less-developed countries, including Ghana. The prevalence of anaemia in Ghana seriously affects the country's social and economic development.

**Aim:** This study, therefore, assessed anaemia in pregnant women attending the Catholic Hospital in the Battor District, Ghana.

**Methods:** A cross-sectional study using a convenient sampling method was conducted from November 2021 to January 2022 among four hundred (400) pregnant women receiving antenatal care at the Catholic Hospital, Battor, in the Volta Region. Sociodemographic (age, marital status, level of education, occupation and religion), knowledge, attitudes, and practices were collected with the aid of a structured questionnaire, and the gestation periods and the number of antenatal (ANC) visits were obtained from the antenatal booklets. Blood samples (3 mL) were collected to determine their anaemia status. SPSS software version 26 and GraphPad Prism 6 were used for the statistical analysis. A *p* value less than 0.050 was considered statistically significant at a 95% confidence interval.

**Results:** Our study found that the prevalence of anaemia among the antenatal attendants was 295 (73.8%). Severe anaemia was highest in the second trimester (60.0%). Pregnant women in their third trimester recorded the highest prevalence of both macrocytic hyperchromic and microcytic hypochromic anaemia (66.7%, 50.0%). The study found that the majority of the pregnant women had satisfactory knowledge of anaemia (38.3%), with only 5.3% having excellent knowledge. Hairdressers had 69% lower odds for anaemia as compared to participants with other occupations (aOR; 0.31, 95% CI; 0.12–0.82, *p* = 0.018) whiles 196 (66.4%) of married women were anaemic.

**Conclusion:** Anaemia remains highly prevalent among pregnant women, with notable variations across trimesters and occupations. Unexpected trends, such as lower odds of anaemia among hairdressers and higher prevalence among married women, highlight the need for further studies into occupational and socio-cultural influences on maternal health.

## 1. Introduction

Anaemia is defined as a decrease in circulating red blood cell or haemoglobin concentration (less than 11.0 g/dL), which reduces the oxygen-carrying capacity of the red blood cells, rendering them incapable of meeting the physiological body requirements [1]. It is a public health concern affecting all ages of the population, with its highest prevalence among children under 5 years of age and pregnant women [2]. Globally, anaemia affects 1.62 billion people (25%), among whom 56 million are pregnant women [3]. Over half (56%) of pregnant women in low- and middle-income countries (LMIC) suffer from anaemia, with the highest rates found in sub-Saharan Africa (57%) as compared to European countries [4]. Anaemia during pregnancy is considered severe when haemoglobin concentration is less than 7.0 g/dL, moderate when haemoglobin falls between 7.0 and 9.9 g/dL, and mild from 10.0 to 11 g/dL [5]. Anaemia, though not a disease, can be either symptomatic or asymptomatic with loss of appetite, tachycardia, dizziness, pallor, headaches, palpitation and easy fatigability [6]. In sub-Saharan Africa, the main causes of anaemia in pregnancy are iron deficiency and malaria, followed by micronutrient deficiencies [7]. Iron deficiency is responsible for about 50% of anaemia in pregnancy in Ghana [8]. Generally, anaemia in pregnant women has been reported to have serious adverse outcomes including high maternal death, impaired mental development in children, increased risk of fetal growth retardation, low birth weight, premature delivery, and perinatal mortality [9]. In Ghana, anaemia is seen as a threat to expectant mothers, with a prevalence of 70% [10]. A study conducted in Adidome Government hospital and Battor Catholic hospital, both in the Volta region of Ghana, reported anaemia prevalence to be above 50% from November 2016 to March 2019 [11].

Though the Ghana government, through the Ministry of Health and the Ghana Health Service, has put in place strategies to reduce anaemia among pregnant women, anaemia remains a leading cause of infant and maternal mortality [12]. Some interventions like the Free Maternal Healthcare Policy (FMHCP), anaemia awareness, nutritional awareness, iron and folic acid (IFA) supplementation and prevention of parasitic infections while promoting insecticide-treated nets (ITNS) usage for pregnant women are attempts to reduce this phenomenon. However, despite this national intervention, anaemia among pregnant women remains alarmingly high. Existing studies in Ghana have predominantly focused on prevalence without adequately exploring the knowledge, attitudes, and practices (KAP) of pregnant women, which are critical for the success of public health interventions. Moreover, there is limited data on the severity and haematological patterns of anaemia across trimesters in this population. This study, therefore, sought to bridge this gap by assessing the KAP regarding anaemia, as well as stratifying its severity and haematological characteristics among pregnant women attending antenatal care at Catholic Hospital, Battor.

## 2. Methodology

### 2.1. Study Design

This was a cross-sectional hospital-based study, conducted at the Catholic Hospital, Battor from November 2021 to January 2022. Some four hundred pregnant women were recruited through a convenient sampling method. Questionnaires were administered to assess the demographic characteristics, KAP of the study participants and blood samples (3 mL) were taken aseptically into an EDTA tube for haemoglobin analysis to determine their anaemia status.

### 2.2. Study Site

Catholic Hospital, Battor is located in the North Tongu district within the Volta Region and serves as a District Hospital. The district is located along the Volta River with a total land size of about 1131.64 sq·km and lies within latitudes 5047′N to 60N and longitude 005′E to 0045′E with a population of about 110,891 according to the 2021 population and housing census by Ghana Statistical Service. The district capital Battor, where the hospital is located is 100 kms from Ho, the Regional Capital of the Volta Region and about 90 kms from Accra, the national capital. This was a single-centered project and Catholic Hospital, Battor has a 262-bed capacity. The total Out Patient Department attendance for 2019 was 95,360. The antenatal clinic recorded 9782 antenatal attendances that same year. In 2020, a total of 74,942 Out Patient Department attendances were recorded and 10,784 antenatal attendances were registered. Catholic Hospital, Battor is strategically located to serve three regions. Greater Accra provides about 60% of the client's base, 30% of the clients come from the Volta Region whiles about 10% are from the Eastern Region. The hospital receives several clients from different countries such as Togo and Bennin, and socio-economic backgrounds, but particularly serves the people of Battor, Mepe, Aveyime and its environs. To reduce maternal mortality in this Catholic Hospital, the hospital has created a social media platform where prayer camps and other sister hospitals are signed onto. For patients who need critical care and are in prayer camps, the hospital gets in touch with the leaders and pastors of these camps, arrangements are then made for the client to be transported to the hospital's facility for treatment to reduce maternal mortality. Despite the outreach programs by the hospital, some clients still patronize the services of these prayer camps due to religious and other reasons.

### 2.3. Study Population

The study population comprised of pregnant women who attended antenatal clinic at the Catholic Hospital, Battor and consented to be part of the study.

### 2.4. Inclusion Criteria

Pregnant women who consented to take part in the study.

### 2.5. Exclusion Criteria

Nonpregnant women and nonconsented pregnant women. Pregnant women who were severely ill or had a history of chronic disorders or bad obstetrics were excluded, as well as those who planned to give birth in other hospitals.

### 2.6. Definition of Anaemia

Anaemia status of pregnant women was determined using WHO guidelines; normal (> 11 g/dL), mild (10–10.99 g/dL), moderate (7.0–9.9 g/dL), and severe (< 7.0 g/dL).

### 2.7. Sample Size Determination

In 2019 for instance, 9782 expectant mothers attended the antenatal clinic in Catholic Hospital, Battor, whiles 10,784 attendances were recorded in 2020. In 2021, the attendance increased to 11,624. The sample size for the study was determined by using the Yamane (1967) sampling size determination formula. At a 95% confidence interval with a margin of error of 0.05, the formula is given as follows:

(1) $$\begin{array}{c} n=\frac{N}{1+{Ne}^{2}}, \end{array}$$

where *n* = sample size, *N* = target population and *e* = level of precision (5%). N is the number of expectant mothers who visited the antenatal clinic in 2020 (9781).

Therefore, *N* = 9,782, hence *n* = 9782/(1 + 9782(0.05)^2^), = 384.27. However, we extrapolated it to 400 to account for potential nonresponse or incomplete data and also to improve the power of statistical analysis.

A convenient sampling technique was employed in this study.

### 2.8. Data Collection Methods

A convenient sampling and structured questionnaire were used to get information on their sociodemographic characteristics (age, marital status, educational status, religion, occupation and gestational period) and the KAP of the pregnant women regarding anaemia in pregnancy. The instrument was guided by existing literature [1, 13], WHO recommendations on maternal anaemia, and adapted to reflect locally relevant practices and beliefs. The knowledge section included items on awareness of anaemia, causes, symptoms, effects, and prevention. Attitudes were assessed using statements regarding the perceived seriousness of anaemia, the importance of iron-rich diets, and ease of preparing such meals. Practices included dietary habits, iron supplementation, deworming, and health-seeking behaviours.

To ensure content validity, the draft questionnaire was reviewed by three experts in haematology, public health, and obstetrics. A pretest was conducted among 30 pregnant women from a nearby antenatal clinic to check for clarity and cultural appropriateness. Based on the feedback, minor modifications were made to improve the wording and flow. Internal consistency reliability was assessed using Cronbach's alpha for the KAP components, yielding 0.72, indicating acceptable reliability.

### 2.9. Sample Collection and Laboratory Analysis

About 3 mL of venous blood was collected aseptically into a labelled EDTA tube by using the standard Venus sample collection method after obtaining consent from each participant for haemoglobin, Mean Corpuscular Volume (MCV), Mean Corpuscular Haemoglobin (MCH), MCH Concentration (MCHC) analysis using Sysmex KX-21N Automated Haematology Analyzer to check their haemoglobin level for anaemia status. The Sysmex KX-21N analyzer underwent regular calibration according to the manufacturer's guidelines. Calibration was performed using standard calibrators, and the instrument was checked daily before use to ensure accurate haemoglobin and red cell indices (MCH, MCV and MCHC) measurement. Additionally, we followed routine quality control procedures, including the use of internal control samples with known haemoglobin and red cell indices concentrations. These control samples were run alongside patient samples to monitor the precision and accuracy of the analyzer. The quality control results were reviewed regularly, and any deviations from the acceptable range led to a re-calibration of the instrument.

### 2.10. Data Management

Data were specially coded to ensure the confidentiality of study subjects and their identity and was stored on a laptop with a password for restricted access. Results and any information concerning the study participants were kept confidential.

### 2.11. Data Analysis

Data from the questionnaire and the laboratory analysis of the haemoglobin were cleaned for completeness and were then analyzed using (SPSS 26 Inc., Chicago, IL, USA) and GraphPad Prism 6. Categorical outcomes were expressed as frequency and proportions. Chi-square test was used to test the association between sociodemographic characteristics and anaemia. Pearson chi-square was used as *p* value. 3 logistic regression models were used to determine factors associated with anaemia in pregnancy. Model 1 adjusted for age, marital status and educational level, model 2 adjusted for religion, occupation, gestational period and overall knowledge level and model 3 adjusted for age, marital status, educational level, religion, occupation, gestational period and overall knowledge level on anaemia. All *p* values < 0.05 was considered statistically significant. Overall knowledge was obtained by scoring the questions that assessed knowledge and the scores were categorized into percentiles; 0–25; poor, 26%–50%; satisfactory, 51%–75%; good, 76%–100%; excellent.

## 3. Results

### 3.1. Sociodemographic Characteristics of Study Participants

A total of 400 participants were involved in this study, with a median age of 29 years (range; 15–46). The largest age group 115 (28.7) was found to be 25–29 years, comprising 28.7% of the study participants. The majority of the participants were married, 283 (70.8%), while educational levels were mainly basic, 231 (57.8%). Christianity was observed to be the dominant religion, 385 (96.3%). In terms of occupation, petty trading was reported as the most common, 149 (37.3%), followed by unemployment, 79 (19.8%). Concerning gestational period, the majority of participants were noted to be in their third trimester 203 (50.7%), with the second trimester being the next most common 145 (36.3%) (Table 1).

### 3.2. Prevalence of Anaemia Among Study Participants

**Figure 1:**
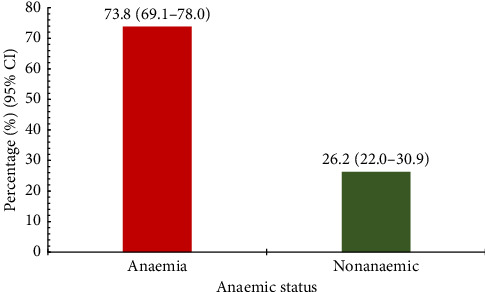
Prevalence of anaemia among study participants.

The majority of the participants were anaemic, 295 (73.8%, 95% CI; 69.1–78.0), whiles the nonanaemic group was 105 (26.2%, 95% CI; 22.0–30.8) of the study participants (Figure 1).

### 3.3. Severity of Anaemia Stratified by Gestational Period

**Figure 2:**
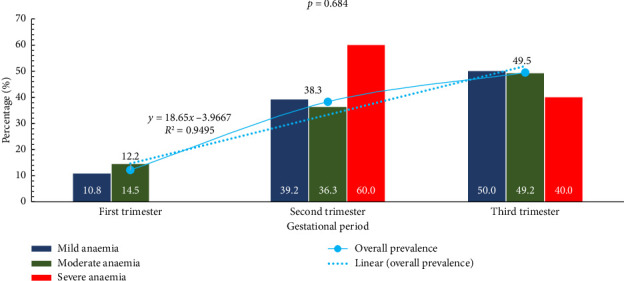
Prevalence of anaemia among study participants. Anaemia status of pregnant women was determined using WHO guidelines into normal (> 11 g/dL), mild (10–10.99 g/dL), moderate (7.0–9.9 g/dL), and severe (< 7.0 g/dL).

Overall, the prevalence of anaemia was found to be increasing as pregnancy progresses across the trimesters (12.2%, 38.3%, 49.5%). The coefficient of determination revealed that 95% of the variations in the prevalence of anaemia could be explained by changes that occurred within the various trimesters. The *p* value of 0.684 is noted at the top of the graph, suggesting the observed differences may not be statistically significant (Figure 2).

### 3.4. Classification of Anaemia Based on Red Cell Indices Stratified by Gestational Period

**Figure 3:**
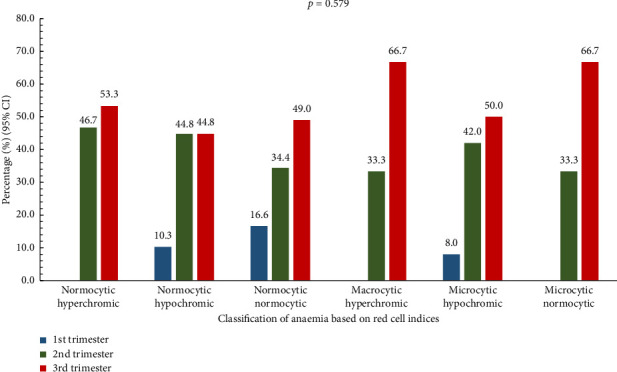
Classification of anaemia based on red cell indices stratified by gestational period.

Normocytic normochromic anaemia was highest in the third trimester (49.0%). Normocytic hyperchromic anaemia was observed to be most prevalent in the third trimester (53.3%), while normocytic hypochromic anaemia was seen to increase from the first (10.3%) to the third (44.8%) trimester. Macrocytic hyperchromic anaemia was found to be highest (66.7%) in the third trimester. Microcytic hypochromic anaemia was noted to be highest in the third trimester (50.0%). It was also observed that microcytic normocytic anaemia was only recorded in the second and third trimesters, with the third trimester being the highest (66.7%). However, these findings were not statistically significant (*p* = 0.579) (Figure 3).

### 3.5. Knowledge of Anaemia by Study Participants

It was found that anaemia had been heard of by 324 (81%) of respondents, though only 126 (31.5%) could define it. Anaemia was recognized as a health problem by 79.8% of participants. The role of vitamin C in iron absorption was known by 174 (43.5%). Regarding symptoms, a pale face was most commonly identified, 255 (37.8%). Hospitals were reported as the primary source of information about anaemia. A lack of iron in food was cited as the main cause 211 (30.1%), while the effects on pregnancy and delivery were noted. For prevention, consuming iron-rich foods was most frequently mentioned 276 (42.1). Overall knowledge levels were categorized, with 153 (38.3%) of participants deemed to have satisfactory knowledge, while excellent knowledge was demonstrated by only 21 (5.3%) (Table 2).

### 3.6. Anaemia Practices by Study Participants

It was found that special local foods believed to boost blood were consumed by 246 (61.5%) of respondents. Regular tea or coffee consumption was reported by 104 (26%) of participants. Iron supplements were taken daily by 340 (85%) of the individuals surveyed. De-worming during pregnancy was practiced by only 98 (24.5%) of respondents. Pica behavior was observed in 86 (21.5%) of participants. When diagnosed with anaemia, iron, vitamin B, and folic acid supplementation was preferred by 282 (70.5%) of respondents, while herbal preparations were favored by 102 (25.5%). In cases of pregnancy-related illness, hospitals were chosen as the primary reporting location by 365 (91.3%) of participants, with prayer camps and herbalists being less frequently selected options (Table 3).

### 3.7. Attitudes of Study Participants Towards Anaemia

Self-awareness regarding anaemia was reported by only 156 (39%) of study participants. The seriousness of anaemia was recognized by 267 (66.8%) of participants, who viewed it as a significant health problem, while 53 (13.3%) considered it normal during pregnancy. The importance of including iron-rich foods in the diet was acknowledged by 329 (82.3%) of participants. Regarding the preparation of iron-rich meals, 275 (68.8%) of participants did not find it difficult, although 49 (12.2%) perceived it as very challenging (Table 4).

### 3.8. Bivariate Logistic Regression of Factors Associated With Anaemia Among Pregnant Women

Marital status was found to be significantly associated with anaemia, with married women showing a higher prevalence 196 (66.4%), followed by single pregnant women 51 (17.3%) with cohabiting pregnant women being the last 48 (16.3%), *p* = 0.002. In model 1 of the bivariate logistic regression, participants with no education had higher odds for anaemia (aOR; 3.10, 95% CI; 1.04–9.27, *p* = 0.043). In model 2, hairdressers had 69% lower odds for anaemia as compared to participants with other occupations (aOR; 0.31, 95% CI; 0.12–0.82, *p* = 0.018). (Table 5).

## 4. Discussion

Our study found a high prevalence of anaemia (73.8%, 95% CI: 69.1–78.0) among pregnant women at Battor Catholic Hospital. This prevalence is significantly higher than the global estimate of 37% of anaemia in pregnant women [14], 40.8% reported by Fondjo and colleagues in a study conducted among pregnant women in their first antenatal care visit in the Volta and Ashanti regions of Ghana [15] and 23.2% by Lebso et al. [16] in a systematic review and meta-analysis in Ethiopia. However, it aligns more closely with a study conducted in the Greater Accra Region in Ghana, where anaemia prevalence was 70.3% among pregnant women [1]. The higher prevalence in the current study might be due to regional variations, differences in study settings, or temporal changes in anaemia prevalence. The study found a significant association between marital status and anaemia, with married women showing the highest prevalence (66.4%). Similarly, a study by Wemakor and colleagues also found anaemia to be more prevalent among married pregnant women (50.3%) [17]. However, our findings and those of Wemakor's contrast with some studies that have found single or unmarried women to be at higher risk of anaemia. For instance, a study in Ethiopia reported that unmarried women had higher odds of anaemia compared to married women [16]. This discrepancy may be due to several context-specific factors, such as increased parity among married women, which is associated with a higher risk of anaemia due to repeated pregnancies and associated nutritional demands. Additionally, access to healthcare, iron supplementation, and dietary practices may differ between marital statuses, influencing anaemia prevalence.

This present study also found that participants with no education had higher odds of anaemia. Similarly, a study in Ethiopia reported that women with no formal education had 2.28 times higher odds of being anaemic compared to those with secondary and above education [18]. A study in Northwest Ethiopia reported that women who could not read and write were 1.9 times more likely to be anaemic compared to those who had secondary and above education [19]. Education can influence health-seeking behaviors, nutritional knowledge, and overall health awareness, which may explain this association. In this present study, hairdressers had 69% lower odds of anaemia compared to other occupations are intriguing and somewhat unexpected. This specific occupation-related finding is not commonly reported in other studies. However, occupation type has been associated with anaemia risk in another study. For instance, a study in Nigeria found that housewives were more likely to be anaemic compared to employed women [20]. Although the finding that hairdressers had significantly lower odds of anaemia compared to other occupational groups may appear unexpected, it is possible that, Hairdressers, particularly those operating in peri-urban areas like Battor, may experience lower client volumes and reduced work-related stress, which allows them more time to rest, maintain better self-care routines, and adhere to antenatal appointments and nutrition guidelines. Furthermore, being in service-oriented roles, they may have greater exposure to health-related conversations with clients or access to media sources that promote awareness of anaemia prevention and healthy practices during pregnancy. While these factors could contribute to their lower anaemia risk, the association should be interpreted with caution and explored further in future studies.

The results in this study showed a clear trend of increasing anaemia prevalence as pregnancy progresses, with 12.2% in the first trimester, 38.3% in the second trimester, and 49.5% in the third trimester. In Ghana, a study in the Sunyani Municipal Hospital found that anaemia prevalence increased with gestational age, with 42.4% in the first trimester, 50.0% in the second trimester, and 54.8% in the third trimester [21]. While the overall trend is similar, the current study shows a more dramatic increase from the first to the second trimester. The sharp increase in severe anaemia cases to 60% in the second trimester, followed by a decrease to 40% in the third trimester, is unusual, though not statistically significant.

Again, though not statistically significant, our study revealed a high prevalence of microcytic hypochromic anaemia in the third trimester (50.0%) is consistent with iron deficiency anaemia, which often worsens as pregnancy progresses. This is similar to findings in Uganda, where iron deficiency was the most common cause of anaemia in pregnancy [22]. On the one hand, the present study found that high awareness of anaemia (81%) among participants is encouraging. However, the low percentage of participants who could define anaemia (31.5%) in the current study indicates a gap between awareness and understanding. The recognition of anaemia as a health problem by 79.8% of participants is similar to findings from a study in India, where 76% of pregnant women considered anaemia as a serious health problem [23]. The knowledge about Vitamin C's role in iron absorption (43.5%) was found to be higher than reported in a study by Onyeneho and colleagues in Nigeria, 28.3% [24].

The consumption of special local foods believed to boost blood (61.5%) in this present study is an interesting finding that reflects cultural practices. This aligns with a study in Ghana, which found that 58.3% of pregnant women consumed local herbs believed to improve blood levels [25]. The present study found an encouragingly high rate of daily iron supplement intake (85%), and higher than reported in some other African countries. A study in Tanzania found that only 58.6% of pregnant women took iron supplements regularly [26]. The low rate of deworming during pregnancy (24.5%) is concerning and lower than recommended. The recognition of anaemia as a serious health problem by 66.8% of participants is positive but slightly lower than some other studies. The aforementioned study in India found that 76% of pregnant women considered anaemia a serious problem [23].

## 5. Conclusion

This study reinforces the persistent high burden of anaemia among pregnant women, particularly in the second and third trimesters. While the general patterns align with previous studies, our findings offer nuanced insights into occupational disparities, specifically, the significantly lower odds of anaemia among hairdressers, which may be attributed to context-specific lifestyle factors in peri-urban areas. Additionally, the disconnect between high anaemia prevalence among married women and prevailing literature suggests the need for further exploration of socio-cultural and household dynamics influencing nutritional health during pregnancy. Though our methodology mirrors established KAP frameworks, this study underscores the importance of integrating occupational and marital context into anaemia risk assessments, and it calls for more qualitative or mixed-methods research to uncover underlying mechanisms driving these associations.

## 6. Limitations

This study has limitations, in that it was conducted in a single hospital-based clinic and used convenience sampling, which prevents generalization of the findings to the entire population. It focused mainly on sociodemographic and did not consider other important factors such as obstetric factors, haematological factors, and dietary factors. Morphological examination was not done to confirm morphological types of anaemia and so such results should be interpreted with caution. This study is subject to potential recall bias, as participants were required to recall past behaviors, symptoms, and sources of information related to anaemia, which may not always be accurate. Social desirability bias may also have influenced some responses, especially on sensitive or behavior-based questions within the KAP sections, as participants may have provided answers, they perceived to be favorable or acceptable rather than reflecting their true beliefs or actions.

## 7. Recommendation

Given the high burden of anaemia observed among pregnant women in this peri-urban setting, we recommend the strengthening of antenatal nutrition education programs with an emphasis on practical dietary guidance and iron supplementation. Health interventions should consider occupational and marital contexts, as these may influence anaemia risk in nuanced ways. Tailored messaging targeting married women and informal sector workers may improve effectiveness. Future research should adopt mixed-method or longitudinal designs to explore the socio-cultural, dietary, and obstetric determinants of anaemia in pregnancy. Additionally, community-based studies with broader representation and inclusion of laboratory-confirmed morphological analysis are encouraged to enhance generalizability and diagnostic accuracy.

## Figures and Tables

**Table 1:** Sociodemographic characteristics of study participants.

Variables	Frequency	Percentage (%)
Total	400	100.0
Age (years)		
Median (minimum-maximum)	29 (15–46)	
15–19	31	7.8
20–24	78	19.5
25–29	115	28.7
30–34	93	23.3
> 35	83	20.8
Marital status		
Single	65	16.3
Married	283	70.8
Cohabiting	52	13.0
Educational level		
Basic	231	57.8
Secondary	101	25.3
Tertiary	32	8.0
None	36	9.0
Religion		
Christian	385	96.3
Muslim	15	3.8
Occupation		
Civil/servants	33	8.3
Hairdresser	31	7.8
Petty trading	149	37.3
Seamstress	41	10.3
Unemployed	79	19.8
Others	67	16.8
Gestational period		
First trimester	52	13.0
Second trimester	145	36.3
Third trimester	203	50.7

**Table 2:** Knowledge of anaemia by study participants.

Variables	Frequency	Percentage
Have you heard about anaemia? (*n* = 400)		100.0
Yes	324	81.0
No	76	19.0
Can you define anaemia? (*n* = 400)		
Yes	126	31.5
No	274	68.7
Is anaemia a health problem? (*n* = 400)		
Yes	319	79.8
No	81	20.3
Do you know vitamin C enhance iron absorption (*n* = 400)		
Yes	174	43.5
No	226	56.5
Do you know the symptoms of anaemia? (*n* = 675)^∗^		
Easy fatigability	182	27.0
Spoon shape nails	46	6.8
Pale face	255	37.8
Breathlessness	57	8.4
Others	53	7.9
Don't know	82	12.1
What is your source of information regarding anaemia? (*n* = 505)^∗^		
Television	72	14.3
Radio	65	12.9
Hospital	273	54.1
Internet	21	4.2
School	66	13.1
None	8	1.6
Do you know the cause of anaemia? (*n* = 702)^∗^		
During pregnancy	73	10.4
Lack of iron in food	211	30.1
Due to any other disease	67	9.5
Due to over bleeding	67	9.5
Others	17	2.4
Don't know	267	38.0
Do you know the effects of anaemia (*n* = 675)^∗^		
Pregnancy	70	10.4
During or after delivery	114	16.9
Complications in delivery	113	16.7
Effects on foetus	77	11.4
Don't know	261	38.7
Others	40	5.9
Do you know about ways to prevent anaemia? (*n* = 655)^∗^		
Consume iron rich foods	276	42.1
Consume vitamins C	106	16.2
Consume iron tablets	169	25.8
Prevention of anaemia caused due to other diseases	31	4.7
Don't know	73	11.1
Overall knowledge		
Poor	128	32.0
Satisfactory	153	38.3
Good	98	24.5
Excellent	21	5.3

^∗^Denotes multiple response.

**Table 3:** Anaemia practices by study participants.

Variables	Frequency	Percentage (%)
Total	400	100.0
Do you feed on any special local food believed to be a blood booster?		
No	154	38.5
Yes	246	61.5
Do you consume tea or coffee regularly?		
No	296	74.0
Yes	104	26.0
Do you frequently take an iron supplement as prescribed?		
Daily	340	85.0
Every other day	16	4.0
Rarely	9	2.3
No	33	8.3
Once a week	2	0.5
Have you taken any de-wormer during your pregnancy?		
No	302	75.5
Yes	98	24.5
Do you have pica (eat clay, dirt)?		
No	314	78.5
Yes	86	21.5
What treatment option do you prefer when diagnosed with anaemia?		
Blood transfusion	16	4.0
Herbal preparation	102	25.5
Iron, vitamin B, folic acid	282	70.5
Where do you report to when you are unwell with pregnancy?		
Hospital	365	91.3
Prayer camp	33	8.3
Herbalist	2	0.4

**Table 4:** Attitudes of study participants towards anaemia.

Variables	Frequency	Percentage (%)
Total	400	100.0
Self-awareness regarding anaemia?		
Aware	156	39.0
Not aware	244	61.0
Attitude regarding the seriousness of anaemia?		
Anaemia is normal in pregnancy	53	13.3
Anaemia is a serious health problem	267	66.8
Anaemia is not a serious health problem	17	4.3
Don't know	63	15.8
Attitude on the importance of including iron-rich foods in the diet.		
Important	329	82.3
Not important	13	3.3
Don't know	58	14.5
Attitude regarding the preparation of iron-rich meals?		
Very difficult	49	12.2
Not difficult	275	68.8
Don't know	76	19.0

**Table 5:** Bivariate logistic regression of factors associated with anaemia among pregnant women.

Variables	Total *n* (%)	Anaemia *n* (%)	*p* value	Model 1 aOR (95% CI)	*p* value	Model 2 aOR (95% CI)	*p* value	Model 3 aOR (95% CI)	*p* value
Total	400 (100.0)	295 (73.8)							
Age									
15–19	31 (7.8)	28 (9.5)		1				1	
20–24	78 (19.5)	59 (20.0)		0.38 (0.09–1.47)	0.162			0.39 (0.09–1.61)	0.194
25–29	115 (28.8)	82 (27.8)	0.235	0.39 (0.09–1.54)	0.179			0.40 (0.09–1.80)	0.229
30–34	93 (23.3)	68 (23.1)		0.45 (0.11–1.83)	0.262			0.45 (0.10–2.06)	0.301
> 35	83 (20.8)	58 (19.7)		0.39 (0.09–1.64)	0.201			0.40 (0.09–1.88)	0.245
Marital status									
Single	65 (16.2)	51 (17.3)		1				1	
Married	283 (70.8)	196 (66.4)	**0.002**	0.69 (0.33–1.48)	0.341			0.66 (0.30–1.46)	0.304
Cohabiting	52 (13.0)	48 (16.3)		3.27 (0.99–10.83)	0.052			2.80 (0.83–9.45)	0.097
Educational level									
Basic	231 (57.8)	169 (57.3)		1				1	
Secondary	101 (25.2)	73 (24.7)	0.138	1.03 (0.60–1.77)	0.913			0.93 (0.53–1.65)	0.814
Tertiary	32 (8.0)	21 (7.1)		0.84 (0.38–1.88)	0.675			0.61 (0.21–1.79)	0.364
None	36 (9.0)	32 (10.8)		3.10 (1.04–9.27)	**0.043**			2.60 (0.85–7.97)	0.094
Religion									
Christian	385 (96.3)	281 (95.3)				0.20 (0.03–1.54)	0.122	0.20 (0.3–1.69)	0.140
Muslim	15 (3.7)	14 (4.7)	0.062			1		1	
Occupation									
Civil servants	33 (8.3)	23 (7.8)				0.48 (0.18–1.27)	0.139	0.90 (0.27–3.03)	0.867
Hairdresser	31 (7.7)	19 (6.4)				0.31 (0.12–0.82)	**0.018**	0.41 (0.15–1.10)	0.077
Petty trading	149 (37.2)	107 (36.3)				0.52 (0.25–1.07)	0.076	0.60 (0.28–1.27)	0.180
Seamstress	41 (10.2)	27 (9.2)	0.112			0.41 (0.17–1.03)	0.058	0.52 (0.20–1.32)	0.168
Unemployed	79 (19.8)	64 (21.7)				0.89 (0.38–2.10)	0.785	0.61 (0.23–1.61)	0.319
Others	67 (16.8)	55 (18.6)				1		1	
Gestational period									
First trimester	52 (13.0)	36 (12.2)				0.89 (0.45–1.78)	0.745	0.82 (0.40–1.68)	0.589
Second trimester	145 (36.2)	113 (38.3)	0.331			1.40 (0.84–2.34)	0.197	1.39 (0.82–2.35)	0.225
Third trimester	203 (50.8)	146 (49.5)				1		1	
Overall knowledge									
Poor	128 (32.0)	89 (30.2)				0.76 (0.25–2.28)	0.622	0.74 (0.24–2.34)	0.611
Satisfactory	153 (38.2)	120 (40.7)	0.355			1.20 (0.40–3.60)	0.750	1.17 (0.38–3.66)	0.783
Good	98 (24.5)	70 (23.7)				0.77 (0.25–2.33)	0.639	0.82 (0.26–2.60)	0.730
Excellent	21 (5.3)	16 (5.4)				1			

*Note:p* value is significant at *p* < 0.05.

Abbreviation: aOR, adjusted odds ratio.

## Data Availability

All relevant data will be made available upon request from the corresponding author.

## Nomenclature

LMIC: Low- and middle-income countries
FMHCP: Free Maternal Healthcare Policy
IFA: Iron and folic acid supplementation
ITNs: Insecticide-treated nets
ANC: Antenal clinic
EDTA: Ethylene diammine tetra-acetic acid

## References

[1] Vikpebah Duneeh R., Ofori Boadu W. I., Tekutey Narh L. (2024). Anaemia during Pregnancy: a Cross-Sectional Study of Antenatal Attendants at the Madina Pentecost Hospital, La Nkwantanang Municipality, Ghana. *Cogent Public Health*.

[2] Siamisang A. B., Gezmu A. M., Slone J. S. (2023). Prevalence and Associated Risk Factors of Anemia Among Hospitalized Children in a Tertiary Level Hospital in Botswana. *Global Pediatric Health*.

[3] Alene K. A., Mohamed D. A. (2013). Prevalence of Anemia and Associated Factors Among Pregnant Women in an Urban Area of Eastern Ethiopia. *Anemia*.

[4] Stephen G., Mgongo M., Hussein Hashim T., Katanga J., Stray-Pedersen B., Msuya S. E. (2018). Anaemia in Pregnancy: Prevalence, Risk Factors, and Adverse Perinatal Outcomes in Northern Tanzania. *Anemia*.

[5] Gebreweld A., Tsegaye A. (2018). Prevalence and Factors Associated with Anemia Among Pregnant Women Attending Antenatal Clinic at St. Paul’s Hospital Millennium Medical College, Addis Ababa, Ethiopia. *Advances in Hematology*.

[6] Tulu B. D., Atomssa E. M., Mengist H. M. (2019). Determinants of Anemia Among Pregnant Women Attending Antenatal Care in Horo Guduru Wollega Zone, West Ethiopia: Unmatched Case-Control Study. *PLoS One*.

[7] Unger H. W., Ashorn P., Cates J. E., Dewey K. G., Rogerson S. J. (2016). Undernutrition and Malaria in Pregnancy: A Dangerous Dyad?. *BMC Medicine*.

[8] Tettegah E., Hormenu T., Ebu-Enyan N. I. (2023). Risk Factors Associated with Anaemia Among Pregnant Women in the Adaklu District, Ghana. *Front Glob Womens Health*.

[9] Varzaru V. B., Eftenoiu A. E., Vlad D. C. (2024). The Influence of Tumor-specific Markers in Breast Cancer on Other Blood Parameters. *Life*.

[10] Asare E. V., Wilson I., Benneh-Akwasi Kuma A. A., Dei-Adomakoh Y., Sey F., Olayemi E. (2018). Burden of Sickle Cell Disease in Ghana: The Korle-Bu Experience. *Advances in Hematology*.

[11] Frempong N. A., Ahiabor C., Anyan W. K. (2023). Malaria, Urogenital Schistosomiasis, and Anaemia in Pregnant Ghanaian Women. *Journal of Parasitology Research*.

[12] Appiah P. K., Nkuah D., Bonchel D. A. (2020). Knowledge of and Adherence to Anaemia Prevention Strategies Among Pregnant Women Attending Antenatal Care Facilities in Juaboso District in Western-North Region, Ghana. *J Pregnancy*.

[13] Aleboko S., Abdulai K., Ayensu J. (2023). Knowledge, Attitudes, and Practices Regarding Anaemia Among Pregnant Women Attending Antenatal Clinic at the University of Cape Coast Hospital in the Cape Coast Metropolis, Ghana. *Integrated Health Research Journal*.

[14] Who (2019). Anaemia. https://www.who.int/news-room/fact-sheets/detail/anaemia.

[15] Fondjo L. A., Addai-Mensah O., Annani-Akollor M. E. (2020). A Multicenter Study of the Prevalence and Risk Factors of Malaria and Anemia Among Pregnant Women at First Antenatal Care Visit in Ghana. *PLoS One*.

[16] Lebso M., Anato A., Loha E. (2017). Prevalence of Anemia and Associated Factors Among Pregnant Women in Southern Ethiopia: A Community Based Cross-Sectional Study. *PLoS One*.

[17] Wemakor A. (2019). Prevalence and Determinants of Anaemia in Pregnant Women Receiving Antenatal Care at a Tertiary Referral Hospital in Northern Ghana. *BMC Pregnancy and Childbirth*.

[18] Woldu B., Enawgaw B., Asrie F., Shiferaw E., Getaneh Z., Melku M. (2020). Prevalence and Associated Factors of Anemia Among Reproductive-Aged Women in Sayint Adjibar Town, Northeast Ethiopia: Community-Based Cross-Sectional Study. *Anemia*.

[19] Asrie F. (2017). Prevalence of Anemia and its Associated Factors Among Pregnant Women Receiving Antenatal Care at Aymiba Health Center, Northwest Ethiopia. *Journal of Blood Medicine*.

[20] Nwizu E. N., Iliyasu Z., Ibrahim S. A., Galadanci H. S. (2011). Socio-demographic and Maternal Factors in Anaemia in Pregnancy at Booking in Kano, Northern Nigeria. *African Journal of Reproductive Health*.

[21] Anlaakuu P., Anto F. (2017). Anaemia in Pregnancy and Associated Factors: a Cross Sectional Study of Antenatal Attendants at the Sunyani Municipal Hospital, Ghana. *BMC Research Notes*.

[22] Ononge S., Campbell O., Mirembe F. (2014). Haemoglobin Status and Predictors of Anaemia Among Pregnant Women in Mpigi, Uganda. *BMC Research Notes*.

[23] Nivedita K., Fatima Shanthini N. (2016). Knowledge, Attitude and Practices of Pregnant Women Regarding Anemia, Iron Rich Diet and Iron Supplements and its Impact on Their Hemoglobin Levels. *International Journal of Reproduction, Contraception, Obstetrics and Gynecology*.

[24] Onyeneho N. G., I’Aronu N., Chukwu N., Agbawodikeizu U. P., Chalupowski M., Subramanian S. V. (2016). Factors Associated with Compliance to Recommended Micronutrients Uptake for Prevention of Anemia during Pregnancy in Urban, Peri-Urban, and Rural Communities in Southeast Nigeria. *Journal of Health, Population and Nutrition*.

[25] Nonterah E. A., Adomolga E., Yidana A. (2019). Descriptive Epidemiology of Anaemia Among Pregnant Women Initiating Antenatal Care in Rural Northern Ghana. *African Journal of Primary Health Care & Family Medicine*.

[26] Ogundipe O., Hoyo C., Stbye T. (2012). Factors Associated with Prenatal Folic Acid and Iron Supplementation Among 21,889 Pregnant Women in Northern Tanzania: A Cross-Sectional Hospital-Based Study. *BMC Public Health*.

